# Collagen scaffold microenvironments modulate cell lineage commitment for differentiation of bone marrow cells into regulatory dendritic cells

**DOI:** 10.1038/srep42049

**Published:** 2017-02-07

**Authors:** Yongxiang Fang, Bin Wang, Yannan Zhao, Zhifeng Xiao, Jing Li, Yi Cui, Sufang Han, Jianshu Wei, Bing Chen, Jin Han, Qingyuan Meng, Xianglin Hou, Jianxun Luo, Jianwu Dai, Zhizhong Jing

**Affiliations:** 1 State Key Laboratory of Veterinary Etiological Biology, Key Laboratory of Veterinary Public Health of Agricultural Ministry, Lanzhou Veterinary Research Institute, Chinese Academy of Agricultural Sciences, Lanzhou 730046, China; 2 State Key Laboratory of Molecular Developmental Biology, Institute of Genetics and Developmental Biology, Chinese Academy of Sciences, Beijing 100190, China; 3 Reproductive and Genetic Center of National Research Institute for Family Planning, Beijing 100191, China

## Abstract

The microenvironment plays a pivotal role for cell survival and functional regulation, and directs the cell fate determination. The biological functions of DCs have been extensively investigated to date. However, the influences of the microenvironment on the differentiation of bone marrow cells (BMCs) into dendritic cells (DCs) are not well defined. Here, we established a 3D collagen scaffold microenvironment to investigate whether such 3D collagen scaffolds could provide a favourable niche for BMCs to differentiate into specialised DCs. We found that BMCs embedded in the 3D collagen scaffold differentiated into a distinct subset of DC, exhibiting high expression of CD11b and low expression of CD11c, co-stimulator (CD40, CD80, CD83, and CD86) and MHC-II molecules compared to those grown in 2D culture. DCs cultured in the 3D collagen scaffold possessed weak antigen uptake ability and inhibited T-cell proliferation *in vitro*; in addition, they exhibited potent immunoregulatory function to alleviate allo-delay type hypersensitivity when transferred *in vivo*. Thus, DCs differentiated in the 3D collagen scaffold were defined as regulatory DCs, indicating that collagen scaffold microenvironments probably play an important role in modulating the lineage commitment of DCs and therefore might be applied as a promising tool for generation of specialised DCs.

Dendritic cells (DCs) are the most effective antigen-presenting cells in the mammalian immune system and are versatile regulators for maintaining immune homeostasis^1^. The routine method for *in vitro* generation of DCs is seeding of bone marrow haematopoietic stem/progenitor cells (BM-HPCs) or monocytes on tissue culture polystyrene (TCPS) or glass dishes with addition of exogenous cytokines, including granulocyte macrophage colony stimulating factor (GM-CSF) or Flt3 ligand (Flt3L)^2^,^3^. Conventional two-dimensional (2D) culture systems have been extensively applied in the preparation of these cells and evaluation of their biological function. However, 2D culture systems are unable to mimic the interactions of the cell-matrix encountered *in vivo*, owing to the lack of certain physical and chemical cues on their flat, rigid, and uniform surface^4^,^5^.

Cell behaviours, including cell survival, proliferation, migration, and differentiation, have been shown to be regulated by microenvironmental features, such as the stiffness, topography, and soluble factors of the extracellular matrix (ECM)^6^,^7^,^8^,^9^. After sensing the physical and chemical cues of the microenvironment, the subsequent cascading responses within cells are triggered, leading to alteration of gene expression and directed cell fate determination. Notably, 3D culture systems based on biomaterial scaffolds are considered to mimic the natural tissue surroundings and promote *in vivo*-like functional and structural development of cells^10^,^11^,^12^,^13^. It has been demonstrated that 3D cultured cells exhibit considerable differences in cellular morphology, phenotype, and biological functions compared to cells cultured under 2D conditions^14^,^15^,^16^,^17^. In addition, our previous studies have shown that 3D culture was able to maintain the pluripotency of stem cells and direct the cell fate determination of mouse embryonic stem cells^14^,^15^.

In the current study, we sought to explore the role of collagen scaffold microenvironments in the differentiation of bone marrow cells (BMCs) into DCs. We established an *in vitro* 3D collagen scaffold microenvironment and investigated whether BMCs in this culture system demonstrated the ability to differentiate into highly specialised populations of DCs.

## Results

### Microstructural features of the collagen scaffold and morphological characteristics of DCs cultured therein

**Figure 1:**
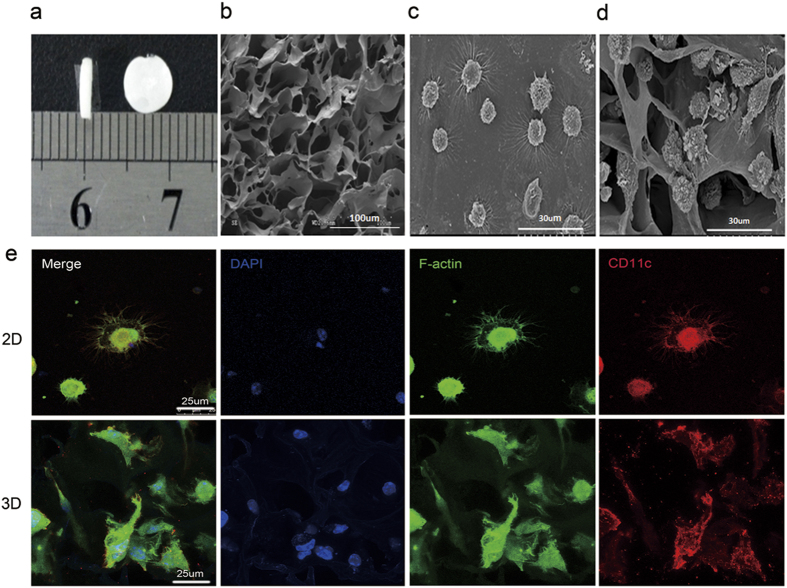
Microstructural features of collagen scaffolds and morphological characteristics of DCs cultured in the 2D and 3D collagen scaffolds. (**a**) Photograph of porous 3D collagen scaffolds. (**b**) SEM image of 3D collagen scaffolds. (**c**) SEM image of DCs differentiated in 2D culture. (**d**) SEM image of DCs differentiated in 3D collagen scaffolds. (**e**) Immunofluorescence staining images of DCs differentiated in 2D and 3D collagen scaffolds under LSCM.

The physical performance of collagen scaffolds was determined using mercury porosimetry. The aperture and porosity of the collagen scaffold were 40.69 um and 96.90%^15^, respectively, and its microstructure as observed by scanning electronic microscopy (SEM) revealed an irregular multiporous structure that was suitable for cell culture *in vitro* (Fig. 1a,b).

Cells cultured in 2D and 3D collagen scaffolds culture were observed by optical microscopy and SEM to investigate their morphological characteristics. After three days of culture, cells cultured in 2D presented a round and irregular shape with a short dendrites. At day 7, most of the cells displayed a typical dendrite appearance and irregular shape under optical microscopy, and presented corona-like-radiating morphology with long and slim dendrites under SEM (Fig. 1c). In comparison, the cells cultured in 3D collagen scaffolds exhibited an irregular shape with short and thick dendrites under SEM (Fig. 1d).

To further elucidate the morphological characteristics of DCs cultured in 2D and 3D collagen scaffolds, the cells at day 7 were stained with fluorescein isothiocyanate (FITC)-phalloidin, and Alexa Flour 594-CD11c, and then imaged using laser scanning confocal microscopy (LSCM). The use of CD11c as a specific marker of murine DCs is widely accepted and F-actin is used to mark the cytoskeleton and the podosomes, which are actin-rich adhesive structures of typical DCs. As shown in Fig. 1e, DCs cultured in 2D displayed corona-like-radiating morphology and an irregular shape with long and slim podosomes, whereas those cultured in 3D collagen scaffolds presented an irregular shape with a small number of short and thick podosomes. The different appearance between 2D- and 3D-cultured DCs indicated that the 3D geometry of the collagen scaffold might induce a change in morphology for these cells.

### Phenotypic characteristic of DCs cultured in 2D and 3D collagen scaffold culture

**Figure 2:**
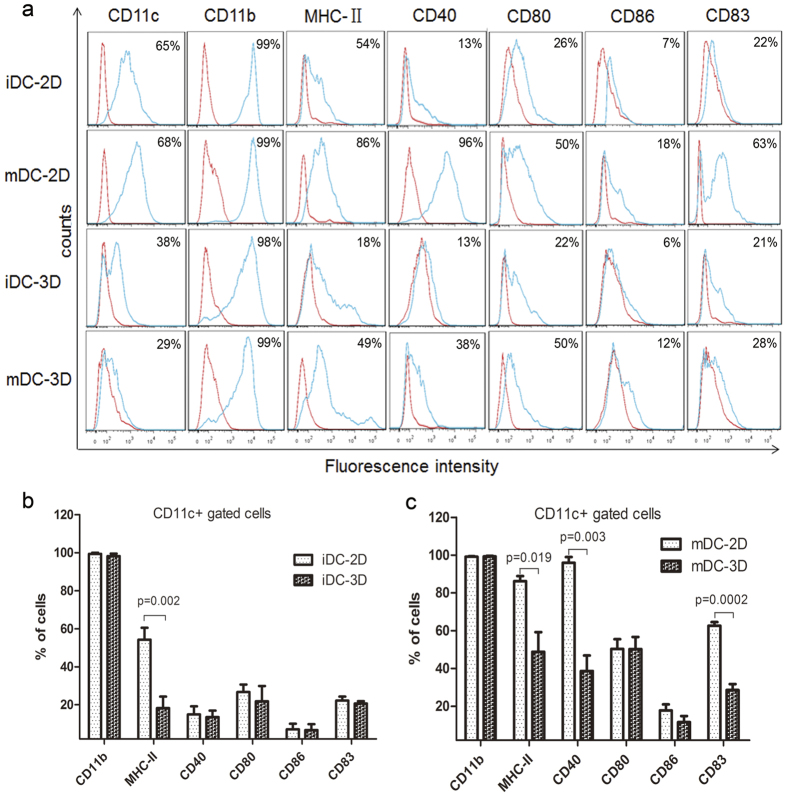
Immunophenotypic analyses of DCs cultured in 2D and 3D collagen scaffolds by FACS. (**a**) Phenotypes of iDCs-2D, mDCs-2D, iDCs-3D, and mDCs-3D. DCs differentiated in 2D and 3D collagen scaffolds were stained using Abs specific for CD11c, CD11b, MHC-II, CD40, CD80, CD86, and CD83 as described in the Materials and Methods. Red lines represent cells stained with isotype-matched control Abs. Statistical analysis of the surface antigen molecules in iDCs-2D and iDCs-3D (**b**) and mDCs-2D and mDCs-3D (**c**). Data are representative of three independent experiments (mean ± SD). **P < 0.01 versus iDCs-2D (**b**). *P < 0.05, **P < 0.01, and ***P < 0.001 versus mDCs-2D (**c**).

To investigate the influence of the 3D collagen scaffold on DCs phenotype, we analysed the expression of CD11c, CD11b, and MHC-II, as well as co-stimulatory molecules including CD40, CD80, CD86 and CD83, in immature (iDCs) and mature (mDCs) DCs using flow cytometry. The expression profile of surface molecules in DCs cultured in 3D collagen scaffolds differed from that in 2D culture. As shown in Fig. 2a, iDCs cultured in both 2D and 3D collagen scaffolds expressed CD11b at extremely high levels, whereas the expression of CD11c and MHC II was lower in iDCs cultured in 3D collagen scaffold than in 2D-cultured iDCs. However, the expression levels of the co-stimulatory molecules in iDCs in the two culture conditions were similar (Fig. 2b).

Upon iDC maturation by stimulation with LPS, the expression levels of MHC-II, CD40, CD80, CD86, and CD83 were significantly increased as determined by flow cytometry. We found that most of the molecules in mDCs cultured in 2D were markedly up-regulated, whereas their expression was only slightly up-regulated in mDCs cultured in 3D collagen scaffolds (Fig. 2c). These findings indicated a stable immature DC-like phenotype for the latter cells, which we termed as CD11b^+^ MHC^lo^ DCs. Overall, these results indicate that the 3D collagen scaffold culture altered the phenotype of DCs derived from BMCs and modulated the lineage commitment of the DCs, resulting in the production of a distinct DC subtype.

### DCs cultured in 3D collagen scaffolds exhibit weak antigen uptake ability

**Figure 3:**
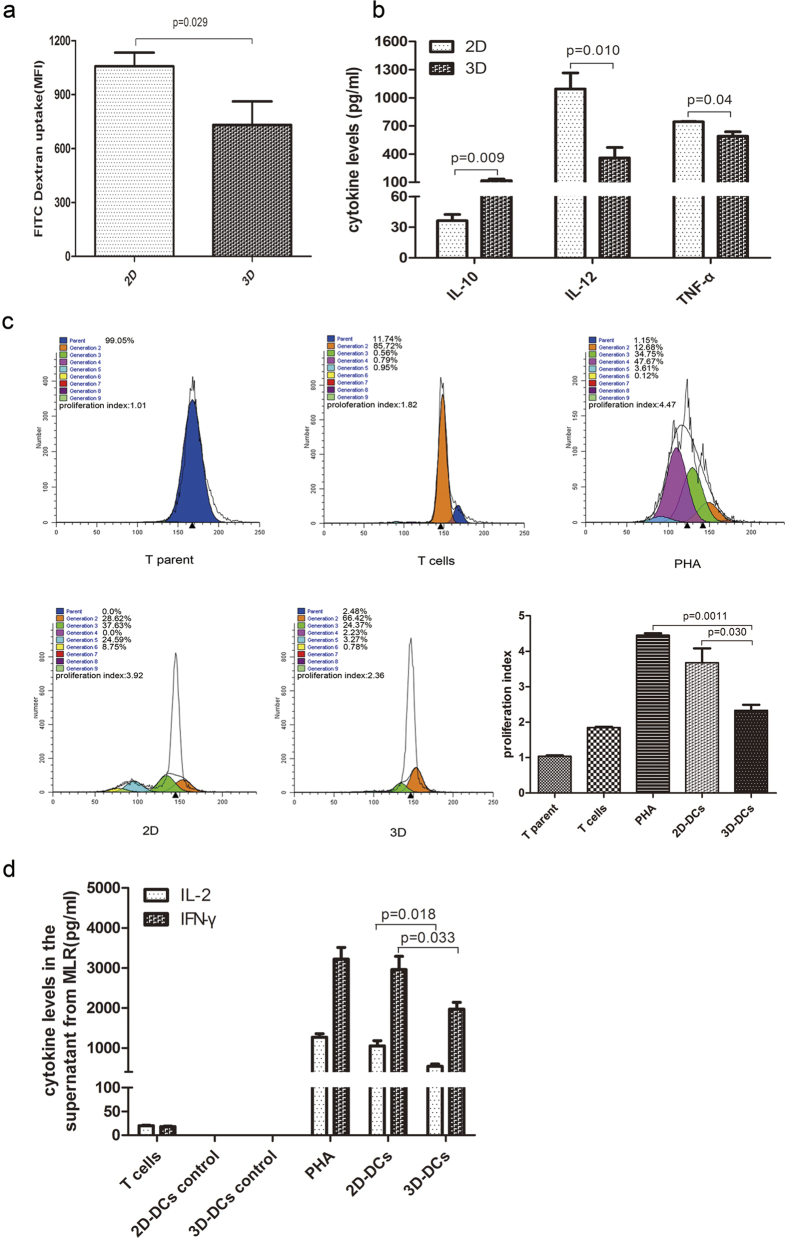
*In vitro* biological functional analysis of cultured DCs. (**a**) Dextran-FITC uptake analysis of iDCs-2D and iDCs-3D. Data are representative of three independent experiments (mean ± SD). *P < 0.05 versus iDCs-2D. (**b**) Cytokine profile analysis of the cell supernatant of mDCs-2D and mDCs-3D. Data are shown as the mean ± SD of three independent experiments. *P < 0.05, **P < 0.01 versus 2D. (**c**) CD4^+^ T cell proliferation analysis of cultured DCs. CD4^+^ T cells from BALB/c mice were labelled with CFSE and co-cultured with cells from C57BL/6 mice from 2D (mDCs-2D) and 3D (mDCs-3D) culture or with T cells that were not stimulated or stimulated with phytohaemagglutinin (PHA) for 4 days. The cells were then harvested and analysed by FACS using ModiFit software. Different colours denote cellular generations. The numbers represent the percentage of different generations among the total cell population. The proliferation index represents the proportion of proliferating cells based on the difference between control and responder cells. Data are representative of three independent experiments (mean ± SD). *P < 0.05 versus mDCs-2D, **P < 0.01 versus PHA. (**d**) Quantification of IL-2 and IFN-γ produced by activated T cells from MLR. Data are shown as the mean ± SD of three independent experiments. *P < 0.05 versus mDCs-2D.

Antigen uptake is a typical characteristic of iDC activity. iDCs cultured in 2D (iDCs-2D) and in 3D collagen scaffold (iDCs-3D) were incubated with FITC-dextran and the intensity of FITC taken up by the iDCs was measured using flow cytometry with the CD11c^+^ gate. We found that the antigen uptake ability in iDCs-3D was much lower than that in iDCs-2D. The mean fluorescence intensity of FITC-dextran uptake was 732 in iDCs-3D, significantly lower than the intensity value of 1058 in iDCs-2D (Fig. 3a). These data suggested that iDCs-3D exhibited a lower antigen uptake activity of compared with iDCs-2D.

### DCs cultured in 3D collagen scaffolds secrete high levels of IL-10

Mature DCs are able to produce pro-inflammatory cytokines, including IL-1β, IL-6, IL-12 and TNF, as well as anti-inflammatory cytokines such as IL-10 and TGF-β. To investigate the cytokine profile of cultured DCs, the cell supernatant from mDCs-2D and mDCs-3D was collected to measure IL-10, IL-12p70, and TNF-α levels using an ELISA kit. As shown in Fig. 3b, mDCs-3D secreted a higher level of IL-10 (approximately 120 pg/ml) and lower levels of IL-12p70 (approximately 1090 pg/ml) and TNF-α (approximately 744 pg/ml) than mDCs-2D. These data indicate that the mDCs cultured in 3D collagen scaffolds produced elevated and reduced amounts of anti-inflammatory and pro-inflammatory cytokines, respectively.

### DCs cultured in 3D collagen scaffolds suppress T-cell proliferation *in vitro*

As previous research has indicated that IL-10 can suppress T cell proliferation^18^, we speculated that the elevated IL-10 expression detected in the specific CD11b^+^ MHC^lo^ DC might indicate a specific biological function in immune regulation. Therefore, we next investigated whether DCs cultured in 3D collagen scaffolds were able to suppress allogeneic T cells *in vitro*. For this, we established a mixed lymphocyte reaction (MLR) assay wherein mDCs-2D and mDCs-3D were each cultured with carboxyfluorescein succinimidyl ester (CFSE)-labelled naïve CD4^+^ T cells for 4 days. Subsequently, T-cell proliferation was measured according to the percentage of cells that lost their CFSE positive signal as determined by flow cytometry. In addition, the cell supernatants from MLR were collected for the determination of cytokine IL-2 and IFN-γ expression by ELISA. As shown in Fig. 3c, the mDCs-2D effectively stimulated allogeneic CD4^+^ T cell proliferation whereas the mDCs-3D exhibited a weaker effect. Consistent with this, we found that mDCs-2D secreted higher levels of activated T cell-related IL-2 and IFN-γ compared with mDCs-3D (Fig. 3d). DCs cultured in 3D collagen scaffolds appeared to suppress T-cell proliferation *in vitro* concomitant with the low levels of IL-2 and IFN-γ expression, in accordance with their high levels of IL-10 expression.

### DCs cultured in 3D collagen scaffolds relieve allo-DTH in C57BL/6 mice

**Figure 4:**
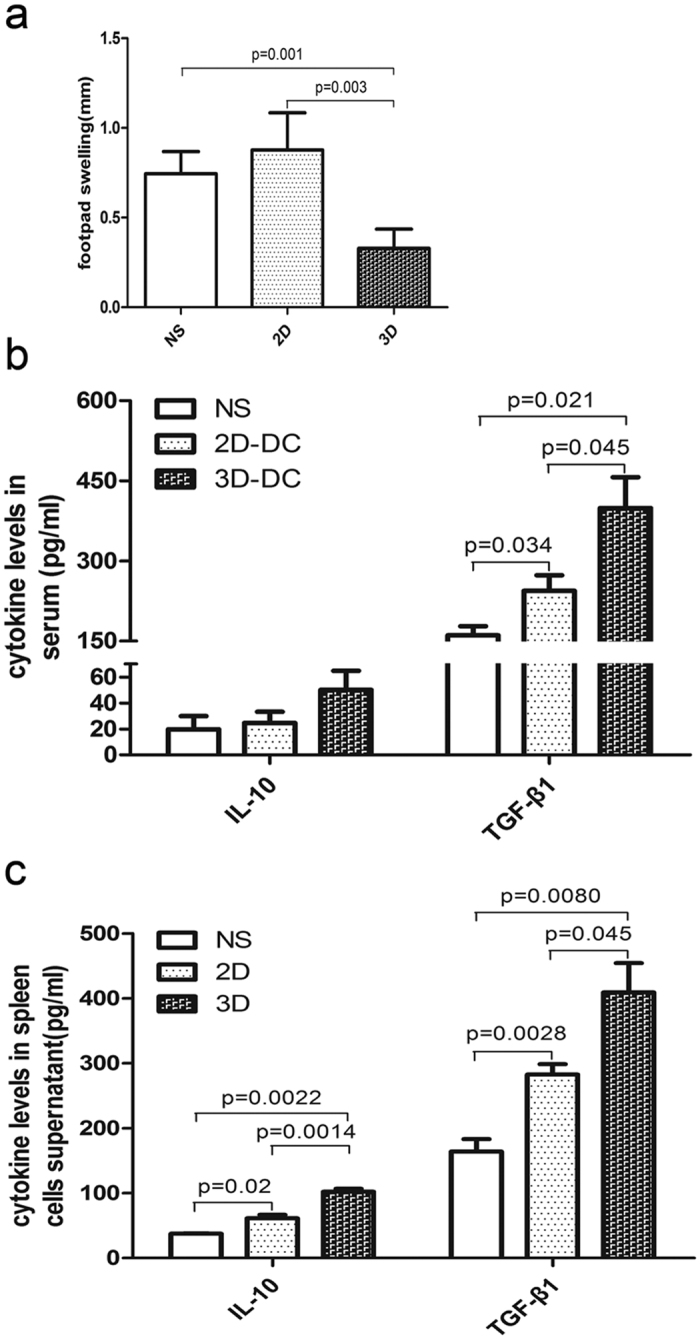
*In vivo* biological functional analysis of differentiated DCs. (**a**) Extent of footpad swelling of the experimental mice (n = 5). **P < 0.01 versus 2D, **P < 0.01 versus NS. (**b**) ELISA of IL-10 and TGF-β1 in the peripheral blood of experimental mice. *P < 0.05 versus mDC-2D and *P < 0.05 versus NS. (**c**) ELISA of IL-10 and TGF-β1 in the cell supernatant of spleen mononuclear cells of experimental mice. Data are shown as the mean ± SD of five samples. For IL-10 expression level analysis, *P < 0.05 versus NS, **P < 0.01 versus mDC-2D, and **P < 0.01 versus NS. For TGF-β1 expression level analysis, *P < 0.05 versus mDC-2D and ** P < 0.01 versus NS.

Based on the above findings, we supposed that DCs cultured in 3D collagen scaffolds may possess the ability to negatively regulate the immune response. To test this hypothesis, we evaluated the immunoregulatory effects of DCs cultured in 3D collagen scaffolds after they are transferred into C57BL/6 mice using the DTH assay. The footpad swelling of C57BL/6 recipient mice immunised with mDCs-2D and mDCs-3D was 0.87 and 0.26 mm, respectively, whereas that of mice treated with saline was 0.74 mm (Fig. 4a). These findings indicate that DCs cultured in 3D collagen scaffolds significantly alleviated allo-DTH in C57BL/6 mice.

It has been demonstrated that one of the suppressive effects of DCs on T cell proliferation is mediated by induction of regulatory T (Treg) cells through the production of anti-inflammatory factors^19^. Therefore, we analysed the proportion of Treg cells among spleen mononuclear cells using flow cytometry and determined the levels of the immunosuppressive cytokines IL-10 and TGF-β1 in peripheral blood and spleen mononuclear cells by ELISA. The proportion of Treg cells in spleen mononuclear cells was low (data not shown) without statistical difference among the three groups. However, the peripheral blood and spleen mononuclear cells from DC-3D-infused mice showed high secreted levels of IL-10 and TGF-β1 (Fig. 4b,c). Together, these results indicate that CD11b^+^ MHC^lo^ DCs suppressed allo-DTH *in vivo*, with high levels of IL-10 and TGF-β1 production.

### 3D collagen scaffold culture alters the gene expression profile in differentiated DCs

**Figure 5:**
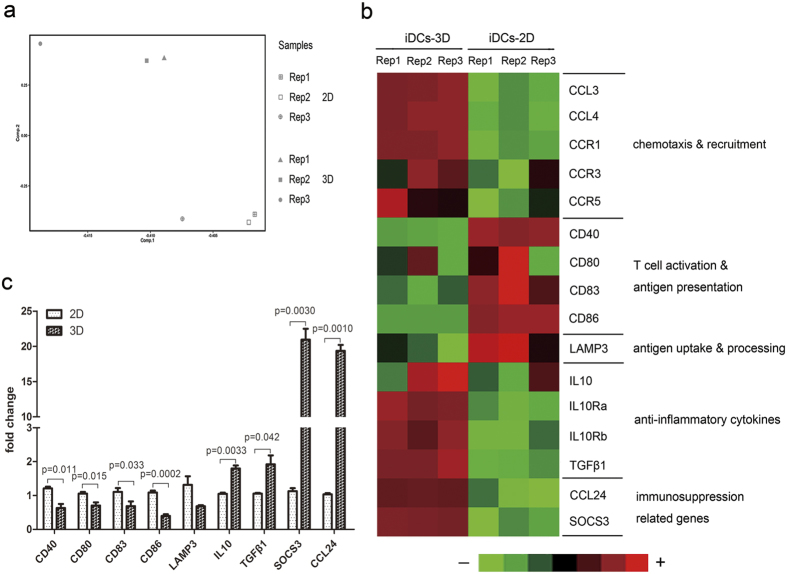
Microarray analyses of iDCs-2D and iDCs-3D. (**a**) Diagram of the principal component analysis of iDCs-2D and iDCs-3D. (**b**) CD molecules and immune-related gene expression analysis of iDCs-2D and iDCs-3D using a transcriptome microarray. Light green represents minimal gene expression, and red shows maximum gene expression as indicated by the legend. (**c**) Quantitative RT-PCR analysis of the expression levels of CD molecules and immune-related genes in iDCs-2D and iDCs-3D. Data are shown as the mean ± SD of three samples. *P < 0.05 versus iDC-2D, **P < 0.01 versus iDC-2D.

To further investigate the biological effects of 3D collagen scaffold culture on DC differentiation and functional regulation, we performed a global transcriptome analysis using the Affymetrix Mouse 430 2.0 expression microarray, revealing a large variation in gene expression profile between iDCs-2D and iDCs-3D. Principal component analysis results indicated that DCs differentiated in 2D and in 3D collagen scaffolds showed significant differences in their gene expression patterns (Fig. 5a). Immune response-related genes are shown in Fig. 5b. Immature DC-related genes such as *Ccl3, Ccl4, Ccr1, Ccr3*, and *Ccr*5 were significantly up-regulated in iDCs-3D compared to iDCs-2D. However, iDCs-3D showed lower expression of T cell activation-related and antigen presentation-related genes such as *Cd40, Cd80, Cd83, Cd86*, as well as the antigen uptake and processing-related gene *Lamp3*, but higher levels of immunosuppression-related genes such as *Il10, Tgfb1, Socs3*, and *Ccl24* than iDCs-2D. By quantitative reverse transcription-polymerase chain reaction (RT-PCR), we further confirmed that the expression levels of *Cd40, Cd80, Cd83, Cd86,* and *Lamp3* were lower in 3D collagen scaffolds than in 2D culture. However, the expression levels of *Il10, Tgfb1, Socs3*, and *Ccl24* were increased in 3D collagen scaffolds (Fig. 5c). These results demonstrate that the gene expression profiles were substantively changed by 3D collagen scaffold culture.

## Discussion

In this study, we cultured BMCs in 3D collagen scaffolds to facilitate their differentiation into a specific subset of DCs, DCregs, and evaluated their functional characteristics. DCs cultured in 3D collagen scaffolds exhibited low expression of MHC and co-stimulatory molecules (CD40, CD80, CD83, and CD86) and demonstrated immunoregulatory functions both *in vitro* and *in vivo*. Thus, our study indicate that the microenvironment plays a critical role in lineage commitment of DCs and that the 3D collagen scaffold described here is suitable for generating specialised DCs from BMCs *in vitro*.

The interactions between cells and the resident microenvironment are pivotal for cellular development, differentiation, and functional regulation *in vivo*. Numerous studies have demonstrated that 3D biomaterial scaffold culture could promote *in vivo*-like functional and structural development of cells^10^,^11^,^12^,^20^. In this analysis, we sought to investigate the role of the microenvironment created by the collagen scaffold in the differentiation of BMCs into DCs. Phenotype analysis using flow cytometry indicated that DCs differentiated in 3D collagen scaffolds expressed lower levels of CD11c, the co-stimulator molecules (CD40, CD80, CD86, and CD83), and MHC molecules than DCs in TCPS/2D culture. Notably, in contrast to DCs-2D, the phenotypes changed only slightly when the DCs cultured in the 3D collagen scaffold were stimulated by LPS, indicating that co-stimulator molecules and MHC molecules were stably expressed on the cell surface at low levels. Thus, DCs differentiated in 3D collagen scaffolds were recognised as a form of the DC mature state rather than representing an immature state of common DCs.

The influence of different biomaterial scaffolds on DC phenotype have previously been investigated^21^. For example, the phenotypic characteristics of iDCs cultured in TCPS/2D and exposed to different biomaterials have been evaluated^22^. Upon exposure of iDCs from human peripheral blood monocytes stimulated with GM-CSF and IL-4 to lactic-co-glycolic acid films, the expression levels of MHC and co-stimulator molecules (CD40, CD80, CD83, and CD86) significantly increased as compared to the control iDCs, as well as DCs cultured on chitosan films expressed these molecules at the high levels. In contrast, DCs cultured on alginate or hyaluronic acid films expressed these molecules at low levels^22^. These results suggest that different biomaterials possess varying immunoregulatory capacities for DC phenotypes. Similarly, the results of the current study demonstrated that the 3D collagen scaffold microenvironment significantly altered the phenotypic features of DCs from BMCs.

Phenotypic characteristics are an important feature for distinguishing among subsets of cells. We noted that the low levels of CD11c, MHC, and co-stimulatory molecule (CD40, CD80, CD83, and CD86) expression observed in DCs differentiated in 3D collagen scaffolds were similar to those exhibited by DCregs. Notably, several types of DCregs derived from different progenitors have been reported to possess immunoregulatory function. For example, CD11b^hi^Ia^lo^ DCregs were derived from bone marrow haematopoietic stem/progenitor cells (BM-HSCs/BM-HPCs) co-cultured with splenic stromal cells and expressed a high level of CD11b and low levels of CD11c, Ia, CD86, CD80, and CD40^23^. Liver DCregs (LRDCs)^24^ differentiated from BM-derived Lin-CD117^+^ progenitors co-cultured with liver stromal cells expressed low levels of CD11c and MHC II, and high level of CD11b. Mesenchymal stem cells (MSC)-DCs were derived from mDCs co-cultured with MSCs and differentiated into a novel DCreg type that expressed low levels of CD11c, Ia, and the co-stimulatory molecules CD80, CD86, CD40, and CD83 as well as high level of CD11b^25^. Sca-1^+^ CD117^−^Lin^−^ mouse embryonic fibroblast (MEF) -MSC-induced DC-like cells^26^ were derived from BM-HPCs co-cultured with MEF-MSCs to differentiate into a distinct DCreg population and expressed a high level of CD11b and low levels of CD11c, Ia, CD80, CD86, and CD40. Therefore, on the basis of phenotypic features, the DCs cultured in 3D collagen scaffolds in the current study were regarded as representing a distinct subset of DCregs, CD11^b+^ MHC^lo^ DCs.

The cytokine mixture of GM-CSF, IL-10, and TGF-β is commonly used to prepare human or murine DCregs *in vitro*^23^. Vitamin D3 and a constructed adenoviral vector coding suppressor of cytokine signalling (SOCS)-1 were also reported to induce the production of DCregs^27^,^28^. GM-CSF is a critical cytokine for generation of human and mouse DCs^29^,^30^. For example, CD11c^hi^Ia^hi^ DCregs were differentiated from BMCs by the addition of a cytokine mixture of murine GM-CSF, murine IL-10, and human TGF-β. These DCregs expressed high levels of CD11c, and MHC molecules with extremely low levels of the co-stimulatory molecules CD40, CD80, and CD86^24^. Notably, in our system, we cultured BMCs stimulated with GM-CSF in the 3D collagen scaffold microenvironment without addition of a DCreg inducer to differentiate the BMCs into DCregs, CD11^b+^ MHC^lo^ DCs. The results therefore indicated that the microenvironment might affect the lineage commitment of DC and that the changes may be dependent on the 3D geometry of the biomaterials.

DCregs plays a pivotal role in the induction of immune tolerance. The main characteristics of DCregs are the secretion of low levels of pro-inflammatory cytokines and high levels of anti-inflammatory cytokines^23^,^24^. In addition, DCregs are able to inhibit T-cell proliferation via anti-inflammatory cytokines^25^,^26^. In the present study, we found that the cell culture supernatant of CD11b^+^ MHC^lo^ DCs contained high level of IL-10, low levels of IL-12 and TNF-α. Furthermore, MLR, which is known as the most representative assay for the measurement of histoincompatibility *in vitro*, has also been employed to evaluate the influence of biomaterials on allogeneic T cell proliferation^31^. In our system, CD11b^+^ MHC^lo^ DCs inhibited the proliferation of T cells in allogeneic MLR assays, with concomitant lower levels of production of IL-2 and IFN-γ, which have been reported^3^ to be crucial cytokines correlated with activated T cells. Together, these results indicate that CD11b^+^ MHC^lo^ DCs comprise a distinct subset of DCregs that possess the capacity to inhibit T cell proliferation *in vitro*, along with high level of IL-10 production.

To examine the immunosuppression capacity of CD11b^+^ MHC^lo^ DCs *in vivo,* we performed an allo-DTH assay. CD11b^+^ MHC^lo^ DCs significantly alleviated allo-DTH in C57BL/6 mice. In addition, the peripheral blood and spleen mononuclear cells from mice infused with CD11b^+^ MHC^lo^ DCs produced high levels of IL-10 and TGF-β1. IL-10, an anti-inflammatory cytokine, has been reported to suppress T-cell mediated immune responses by the down-regulation of MHC, co-stimulatory molecules, and intercellular adhesion molecules-1^18^,^19^,^32^. It can also reduce the production of pro-inflammatory cytokines, such as TNF-a, which possess direct stimulatory function on antigen-presenting cells (APCs)^33^,^34^. Furthermore, IL-10-modulated DCs have been employed as the most prominent tolerogenic DC population for clinical application^35^. TGF-β1 has also been confirmed as a crucial immunoregulatory cytokine in the regulation of T cell-mediated immune responses^36^,^37^. TGF-β can down-regulate proinflammatory cytokines such as IL-12, TNF-α and IFN-α, and increase the production of anti-inflammatory cytokines, including TGF-β itself^38^,^39^,^40^,^41^. Additionally, TGF-β can inhibit the expression of co-stimulatory molecules, such as, CD80, CD83 and CD86, as well as MHC molecules^42^,^43^,^44^. TGF-β, alone or in combination with IL-10, is also capable of inducing tolerogenic DCs. Therefore, on the basis of our data, we proposed that the IL-10 and TGF-β secreted from CD11b^+^ MHC^lo^ DCs may be partly involved in the development of DCregs from BMCs and may enable them to negatively regulate the immune response *in vitro* and *in vivo*.

To further elucidate the mechanism of 3D collagen scaffold microenvironment mediated BMC differentiation into DCs, we performed global transcriptome analysis using a microarray. The mRNAs of the co-stimulatory molecules *Cd40, Cd80, Cd83,* and *Cd86* were down-regulated in iDCs-3D compared to 2D culture. Similarly, antigen uptake and the processing-related gene *Lamp3* were also down-regulated in iDCs-3D. However, the mRNAs for the cytokine immunosuppressive molecules *Il-10* and *Tgf-β1* and the immunosuppression-related molecules *Socs3* and *Ccl24* were up-regulated in iDCs-3D compared to 2D culture. Similar results were observed from quantitative RT-PCR. These results indicate that 3D collagen scaffold culture significantly alters the gene expression profile of DCs derived from BMCs, further supporting the biological functional results.

Cell development and differentiation in natural tissue occur in the context of the microenvironment. Different microenvironments support different types of cell differentiation via physical and chemical signals. After sensing these microenvironmental cues, cascading responses within cells are activated, leading to the alteration of cell behaviour, such as gene expression and cell fate determination^45^. In particular, some previous studies have demonstrated that the geometry and specific topography of biomaterials influence cell behaviour^15^,^46^,^47^. Therefore, in our works, we proposed that the spatial pattern of collagen scaffolds may have facilitated the development of CD11b^+^ MHC^lo^ DCregs from BMCs in the 3D collagen microenvironment. Furthermore, the high levels of production of IL-10 and TGF-β1, factors related to tolerogenic DC production by the resultant CD11b^+^ MHC^lo^ DCregs and their potent immunoregulatory function *in vitro* and *in vivo*, indicated that soluble factors might also be involved in the development of these cells in the 3D collagen microenvironment.

In conclusion, our results demonstrate that the 3D collagen scaffold microenvironment could modulate the lineage commitment of DCs and provide a suitable niche for certain specialised DC development. 3D collagen scaffold culture may therefore represent a promising tool to prepare DCregs for clinical application in autoimmune diseases and for transplantation. However, the mechanisms underlying the effect of the collagen scaffold microenvironment on the development of DCregs have not yet been fully clarified. Therefore, future investigation is required to elucidate the biological effects and definitive mechanism of collagen scaffold microenvironment culture on DCreg development. In addition, the mechanisms by which CD11b^+^ MHC^lo^ DCs negatively regulate the immune response need to be further clarified as well.

## Methods

### Preparation of collagen scaffolds

Collagen type I membrane derived from bovine tendon was purchased from Zhenghai Biotechnology Ltd (Shandong, China). Collagen scaffolds for experiments were prepared according to a published protocol^15^. The physical performance of the prepared collagen scaffolds was determined by mercury porosimetry.

The collagen scaffold slices were immersed in 100 μg/ml poly-D-lysine (PDL) at 37 °C for 2 h, and then washed with Dulbecco’s phosphate buffered saline (DPBS) three times for future application.

### Ethics statements

All animal experimental procedures were approved by the institutional review board of the Institute of Genetics and Developmental Biology, Chinese Academy of Sciences, and performed according to the Chinese Ministry of Public Health (CMPH) Guide for the care and use of laboratory animals.

### Generation of murine bone marrow-derived DCs

BM-derived DCs were prepared from BMCs of C57BL/6 mice. Briefly, BMCs were extracted from tibias and femurs by repeat pipetting and the cell suspensions were passed through a 70-μm nylon mesh to remove cell debris. Red blood cells were removed with red cell lysis buffer (Tiangen Biotech Co., Ltd, China). The BMCs were then harvested and plated on 2D culture dishes and collagen scaffolds, and cultured with DC-medium: RPMI 1640 medium containing 10% heat-inactivated foetal calf serum (Gibco, Gaithersburg, MD), 100 U/ml penicillin, 100 mg/ml streptomycin (Gibco), 0.1 mM β-mercaptoethanol (Gibco) and 20 ng/ml recombinant mouse GM-CSF (rmGM-CSF, R&D Systems, Minneapolis, MN).

For DC 3D collagen scaffold culture, BMCs at a concentration of 3 × 10^5^ cells in 30 μl medium were seeded onto a collagen scaffold slice. After 4 h, DC culture medium was added to cover the collagen scaffold slices and the cultures were incubated for 9 days. Half of the culture medium was replaced in the DC culture every 3 days.

For DC 2D culture, BMCs were seeded at the same cell number used for the 3D collagen scaffold on flat-bottomed wells of 6-well culture plates at 37 °C with 5% CO_2_ for 9 days. iDCs cultured in 2D and in 3D collagen scaffolds were matured by stimulation with 500 ng/ml LPS (Sigma, St. Louis, MO) for the last 2 days.

DCs cultured in 2D and in 3D collagen scaffold slices were recovered after the designated days of incubation by adding an appropriate volume of 0.25% trypsin with 1 mM ethylenediaminetetraacetic acid solution at 37 °C for 5 min. Cells were then collected for subsequent analysis.

### SEM analysis

The morphology of DCs cultured in 2D and in 3D collagen scaffolds for 7 days was observed by SEM (S-3000N; Hitachi, Tokyo, Japan). Briefly, DCs cultured in 2D were recovered and plated on PDL-coated glass cover slips. DCs cultured in 3D collagen scaffolds and the PDL-coated glass cover slips were then washed gently three times with cold DPBS and fixed with cold 4% paraformaldehyde solution for 12 h at 4 °C. Thereafter, the samples were dehydrated, critical-point-dried, and sputter-coated with gold platinum prior to SEM imaging.

### Immunofluorescence staining

On day 7 of culture, samples of 2D-DCs and 3D-DCs were prepared as described above. Samples then were washed gently with DPBS and fixed in 4% paraformaldehyde for 4 h at 4 °C. The primary antibody used in this study was a purified hamster anti-mouse CD11c antibody (1:50, BD Biosciences, San Jose, CA). The secondary antibody was an Alexa Flour 594-conjugated Affinipure Goat anti-Armenian hamster IgG (H+L) (1:100, Jackson ImmunoResearch Laboratories, Inc., Bar Harbor, ME). The nuclei were counterstained with DAPI (1:800, Molecular Probes, Eugene, USA). The actin filaments (F-actin) were stained by FITC-phalloidin (1:20, Sigma). Fluorescence images were observed on a Leica TCS SP8 LSCM (Leica Microsystems, Wetzlar, Germany).

### Phenotype analysis of DCs differentiated in the collagen scaffold

The phenotype of immature and mature DCs cultured in 2D and 3D collagen scaffolds was analysed by fluorescence-activated cell sorting (FACS Aria II, BD) after staining with the following monoclonal antibodies (mAbs), according to the manufacturer’s protocols (BD Pharmingen, Franklin Lakes, NJ): FITC-labelled anti-CD11b, APC-labelled anti-CD11c, FITC-labelled anti-CD11c, PE-labelled anti-CD40, APC-labelled anti-CD80, PE-labelled anti-CD83, PE-Cy7-labelled-anti-CD86 and PerCP-Cy5.5-labelled anti-I-A/I-E. Non-specific staining was blocked with purified rat anti-mouse CD16/CD32 (mouse BD Fc Block) (BD Pharmingen). The isotype controls were stained using the respective antibody. The expression levels of the surface markers were analysed with BD FACSDiva Software v6.1.3 and FlowJo 7.6 (FlowJo Llc, Ashland, USA).

### Determination of DCs phagocytosis

DCs were recovered after the designated number of days, washed three times with DPBS, stained with APC-CD11c (BD Pharmingen) for 30 min at 4 °C, and washed three times with DPBS. The samples then were treated with dextran labelled with FITC (FITC-dextran) (molecular weight 40,000 Da, Sigma) solution (1 mg/ml) for 60 min at 37 °C, and the controls were prepared under the same condition at 4 °C. Thereafter, the samples were washed three times with cold DPBS to stop phagocytosis. At least 10, 000 CD11c^+^ cells per sample were scanned by flow cytometry (FACS Aria II). To accurately account for internalised particles, the mean fluorescence intensity of samples incubated at 4 °C was subtracted from the mean fluorescence intensity of samples incubated at 37 °C by using their geometric means (Gm) (Gm37 °C − Gm4 °C = Gm of phagocytosed particles). Each experiment was performed in triplicate.

### MLR analysis

Murine Naïve CD4^+^ T cells were isolated and purified from splenocytes using a naïve CD4^+^ T cell isolation kit (Miltenyi Biotec, Bergisch-Gladbach, Germany) according to the manufacturer’s instructions.

For CFSE labelling, CFSE (Sigma) solution was added to naïve CD4^+^ T cells to a final concentration of 3 μM, followed by incubation for 10 min at 37 °C, blocking with RPMI/10% FBS solution on ice for 5 min, and washing of the cells. The cells were resuspended at the desired cell concentration and used as responder cells in the MLR assay. The small fraction of CFSE-labelled naïve CD4^+^ T cells was fixed by 4% formaldehyde and used as parent cells.

For MLR experiments, mDCs-2D and mDCs-3D derived from C57BL/6 mice, which were used as stimulator cells, were recovered and treated with mitomycin C (25 mg/mL) (Roche Applied Biosystems, Foster City, CA) for 3 h at 37 °C, and the cells were extensively washed three times with DPBS. The allogenic CFSE-labelled naïve CD4^+^ T cells were cultured with mDCs-2D and mDCs-3D on 96-well U-bottom plates at a 10:1 T-cell:DC ratio. PHA-stimulated or non-stimulated naïve CD4^+^ T cells were plated at the same concentration and used as a positive or negative control. On day 4, the cells were harvested and stained with PE-CD4 (BD Pharmingen). T cell proliferation was analysed using flow cytometry as described^48^. FACS data were analysed using the Proliferation Wizard module of the ModFit software (Verity Software House, Topsham, USA). After incubation, the cell culture supernatants were collected to detect IL-2 and IFN-γ using ELISA kits (eBioscience, San Diego, CA). Each experiment was performed in triplicate.

### Cytokine quantification

Mature DC (mDC) culture supernatants were collected at day 9 of DC culture in 2D and 3D collagen scaffolds, and analysed for IL-12 and IL-10 (eBioscience, USA), TNF-α (Neobioscience technology, China) using ELISA kits.

### *In vivo* allo-DTH assay

mDCs-2D and mDCs-3D derived from C57BL/6 mice were harvested as described previously and injected (3 × 10^6^ cells/mouse) into the C57BL/6 recipient mouse abdomen on days −6, −4, and 0. Then, splenocytes isolated from BALB/c mice were injected into the dorsal subcutaneous tissue of C57BL/6 recipient mice (1 × 10^7^ cells/mouse) on days 0 and 3. On day 7, C57BL/6 recipient mice were challenged by injection of BALB/c splenocytes into the right hind footpad (1 × 10^7^ cells/mouse). The left hind footpad of each recipient mouse was injected with the same amount of normal saline (NS) as a control. The negative control mice were injected with the same amount of NS. At 24 h after injection, the footpad thickness was measured using a micrometer. The extent of footpad swelling was measured by subtracting the baseline thickness of the left footpad from the thickness of the right footpad. The peripheral blood of experimental mice was collected to determine the levels of IL-10 and TGF-β1 using ELISA kits.

To determine the proportion of Treg cells, the splenocytes of experimental mice were isolated using Ficoll, and stained with FITC-labelled anti-CD4 (BD Pharmingen), APC-labelled anti-CD25 (BD Pharmingen), and PE-labelled anti-Foxp3 (BD Pharmingen). The proportion of Treg cells was analysed with BD FACSDiva Software v6.1.3.

### Microarray assay

On day 7, gene expression profiles of the DC culture in 2D and in 3D collagen scaffolds were analysed using the Affymetrix Mouse 430 2.0 expression microarray. The microarray assay was performed by a service provider (Shanghai Biotechnology Corporation, SBC).

### Quantitative RT-PCR

Power SYBR Green RT-PCR Kit (Invitrogen) and Bio-RAD CFX96 Real-Time System (Bio-RAD) were used for quantitative RT-PCR analysis as described^14^. Data were normalised to the reference gene GAPDH for each cDNA sample. The primers used are listed in Table 1.

### Statistical analysis

Students’*t*-test was used to compare means values of two samples. A value of *P* < 0.05 was considered as statistical significance. All experiments were performed at least three times. Each experiment was performed in triplicate. Data were shown as means ± SD.

## Additional Information

**How to cite this article**: Fang, Y. *et al*. Collagen scaffold microenvironments modulate cell lineage commitment for differentiation of bone marrow cells into regulatory dendritic cells. *Sci. Rep.* **7**, 42049; doi: 10.1038/srep42049 (2017).

**Publisher's note:** Springer Nature remains neutral with regard to jurisdictional claims in published maps and institutional affiliations.

## Supplementary Material

Supplementary Information

## Figures and Tables

**Table 1:** List of gene-specific primers for RT-PCR.

Gene name	Primer sequences (5′-3′)
GAPDH	Forward: AGGTCGGTGTGAACGGATTTG
	Reverse: TGTAGACCATGTAGTTGAGGTCA
CD40	Forward: TGTCATCTGTGAAAAGGTGGTCReverse: ACTGGAGCAGCGGTGTTATG
CD80	Forward: ACCCCCAACATAACTGAGTCT
	Reverse: TTCCAACCAAGAGAAGCGAGG
CD83	Forward: CGCAGCTCTCCTATGCAGTG
	Reverse: GTGTTTTGGATCGTCAGGGAATA
CD86	Forward: CTGGACTCTACGACTTCACAATG
	Reverse: AGTTGGCGATCACTGACAGTT
LAMP3	Forward: TCCAAAAGCCAGAGGCTATCT
	Reverse: ACTGGGGTTACTGTTTTCATTGT
IL10	Forward: GCTCTTACTGACTGGCATGAG
	Reverse: CGCAGCTCTAGGAGCATGTG
TGF-β1	Forward: CTCCCGTGGCTTCTAGTGC
	Reverse: GCCTTAGTTTGGACAGGATCTG
SOCS3	Forward: ATGGTCACCCACAGCAAGTTT
	Reverse: TCCAGTAGAATCCGCTCTCCT
CCL24	Forward: ATTCTGTGACCATCCCCTCAT
	Reverse: TGTATGTGCCTCTGAACCCAC
