# Supplementary material for: Collagen scaffold microenvironments modulate cell lineage commitment for differentiation of bone marrow cells into regulatory dendritic cells
Source: Sci Rep. 2017 Feb 7;7:42049. doi: 10.1038/srep42049 (PMC5294561; doi:10.1038/srep42049)

**Collagen scaffold microenvironments modulate cell lineage commitment for differentiation of bone marrow cells into regulatory dendritic cells**

Yongxiang Fang ^a^, Bin Wang ^b^, Yannan Zhao ^b^, Zhifeng Xiao ^b^, Jing Li ^b^, Yi Cui ^b,c^, Sufang Han ^b^, Jianshu Wei ^b^, Bing Chen ^b^, Jin Han ^b^, Qingyuan Meng ^b^, Xianglin Hou ^b^, Jianxun Luo ^a^, Jianwu Dai ^b,*^, and Zhizhong Jing ^a,*^

^a^ State Key Laboratory of Veterinary Etiological Biology, Key Laboratory of Veterinary Public Health of Agricultural Ministry, Lanzhou Veterinary Research Institute, Chinese Academy of Agricultural Sciences, Lanzhou 730046, China.

^b^ State Key Laboratory of Molecular Developmental Biology, Institute of Genetics and Developmental Biology, Chinese Academy of Sciences, Beijing 100190, China

^c^ Reproductive and Genetic Center of National Research Institute for Family Planning, Beijing 100191, China.

*Correspondence authors:

Jianwu Dai, PhD., Professor, Institute of Genetics and Developmental Biology, Chinese Academy of Sciences, 3 Nanyitiao, Zhongguancun, Beijing 100190, China. 86-010-82614426 (phone/fax), E-mail: [jwdai@genetics.ac.cn](mailto:jwdai@genetics.ac.cn);

Zhizhong Jing, PhD., Professor, Lanzhou Veterinary Research Institute, Chinese Academy of Agricultural Sciences, xujiaping 1, Lanzhou, Gansu, 730046, China. 86-931-8341979 (phone), 86-931-8340977 (fax), E-mail: [jingzhizhong@caas.cn](mailto:jingzhizhong@caas.cn);

**Supplementary Figure legends**

**Figure S1. Immunophenotypic analyses of DCs cultured in 2D and 2D collagen-coating by FACS.**

Phenotypes of iDCs-2D, iDCs-2D-collagen-coating, mDCs-2D, and mDCs-2D-collagen-coating. 2D culture dishes were coated with 0.25% collagen type I solution at 37°C for 1 h, and then washed with Dulbecco’s phosphate buffered saline (DPBS) three times for future application. DCs differentiated in 2D and 2D collagen-coating were stained using Abs specific for CD11c, MHC-II, CD40, CD80, CD86, and CD83 as described in the Materials and Methods.


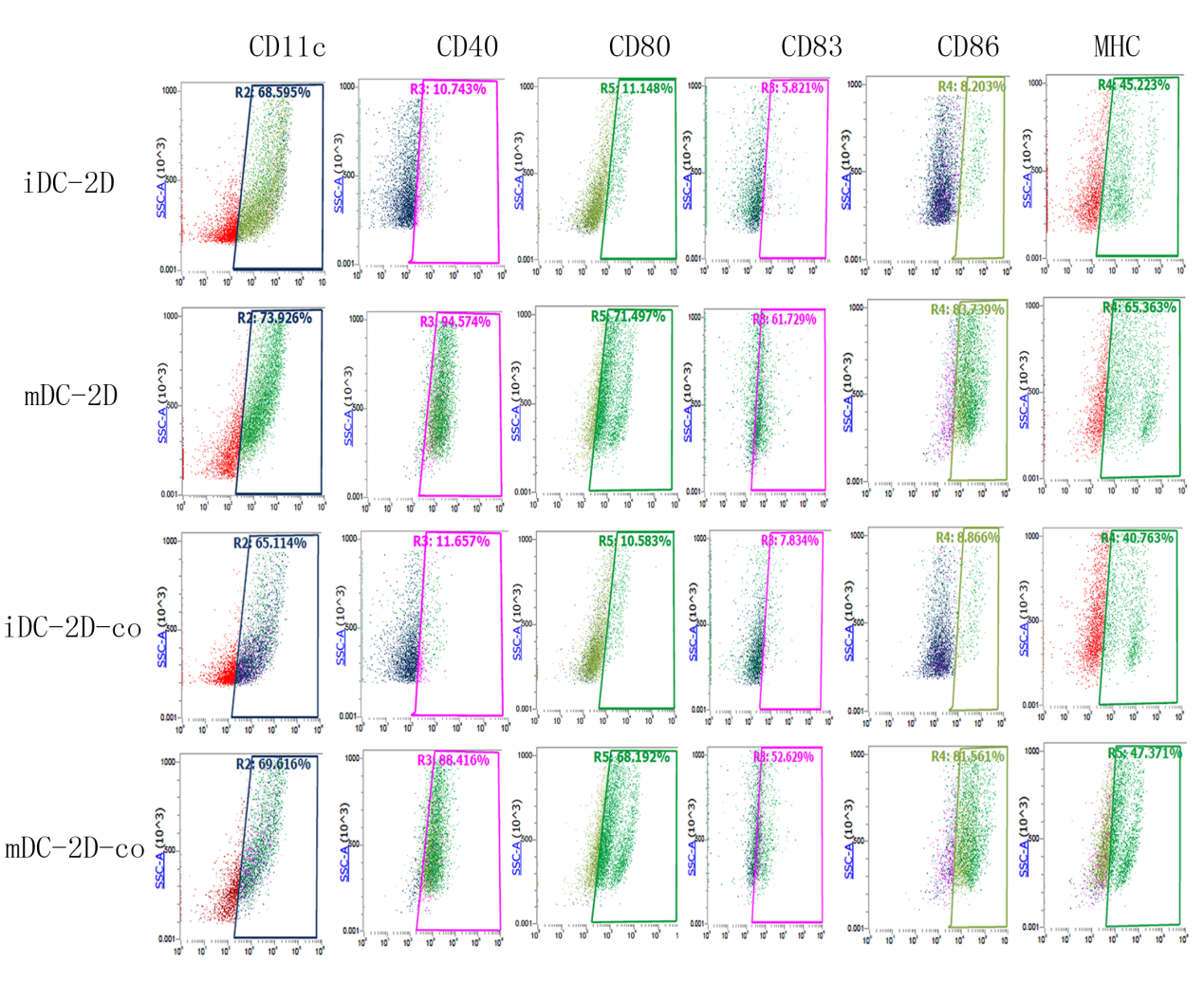

Supplement: Supplementary Information [file srep42049-s1.docx]
